# Mast Cell Modulation of B Cell Responses: An Under-Appreciated Partnership in Host Defence

**DOI:** 10.3389/fimmu.2021.718499

**Published:** 2021-09-10

**Authors:** Alejandro M. Palma, Mark R. Hanes, Jean S. Marshall

**Affiliations:** ^1^IWK Health Centre and Department of Pediatrics, Dalhousie University, Halifax, NS, Canada; ^2^Department of Pathology, Dalhousie University, Halifax, NS, Canada; ^3^Beatrice Hunter Cancer Research Institute, Halifax, NS, Canada; ^4^Department of Microbiology and Immunology, Dalhousie University, Halifax, NS, Canada

**Keywords:** infection, regulatory B cells, allergy, inflammation, CD40

## Abstract

Mast cells are well known to be activated *via* cross-linking of immunoglobulins bound to surface receptors. They are also recognized as key initiators and regulators of both innate and adaptive immune responses against pathogens, especially in the skin and mucosal surfaces. Substantial attention has been given to the role of mast cells in regulating T cell function either directly or indirectly through actions on dendritic cells. In contrast, the ability of mast cells to modify B cell responses has been less explored. Several lines of evidence suggest that mast cells can greatly modify B cell generation and activities. Mast cells co-localise with B cells in many tissue settings and produce substantial amounts of cytokines, such as IL-6, with profound impacts on B cell development, class-switch recombination events, and subsequent antibody production. Mast cells have also been suggested to modulate the development and functions of regulatory B cells. In this review, we discuss the critical impacts of mast cells on B cells using information from both clinical and laboratory studies and consider the implications of these findings on the host response to infections.

## Introduction

The ability of mast cells to aid in the initiation and regulation of acquired immune responses has been demonstrated by multiple authors (1–6). As key resident sentinel cells in the skin and at mucosal surfaces, capable of detecting pathogens and tissue damage, mast cells are often one of the first cell types to be activated on pathogen invasion, tissue damage, or infection. Initial responses to bacterial pathogens often result in the production of an NF-κB-dependent cytokine cascade that includes the production of TNF-α, IL-1β and IL-6, as well as other cytokines and regulatory factors. The balance of mediators produced varies considerably depending on the tissue site and stimulus (2, 7–10). These can include immunomodulatory cytokines such as IL-10, as well as IL-1RA and a wide variety of potent chemokines which recruit appropriate effector cells. In response to several viral infections the production of chemokines, along with type 1 interferons (IFN) represent the predominant mast cell response and leads to the recruitment of NK cells and CD56+ T cells (11–20). Mast cells also respond to tissue damage *via* responses to alarmins, such as IL-33, subsequently giving rise to a further unique pattern of mediators including IL-13 and IL-5 (21–23). While degranulation is induced by certain stimuli, such as nematode parasites and select bacteria, mediator production often occurs in its absence. Lipid mediators are also selectively produced in response to many infections and contribute to cell recruitment and vascular changes. This highly regulated and co-ordinated mast cell response can aid in the mobilisation of dendritic cells that subsequently migrate to draining lymph nodes (2, 7, 24–27), the recruitment of effector cells, and the initiation of an optimal acquired immune response including the production of neutralising antibodies.

In many cases it has been assumed that the interaction of mast cells and B cells is important, but not co-ordinated at the tissue level. Mast cells promote the overall initiation of antibody responses and at the same time mast cells are guided and enhanced in their responses by IgE or IgG subclasses bound to Fc receptors on their surface. However, increasing evidence suggests that the relationship between mast cells and B cells is much deeper and more complex, providing potential opportunities for therapeutic intervention. In this review we have selected just some of these proven and potential interactions to highlight and illustrate the complexity and importance of the mast cell-B cell relationship.

## Receptor-Ligand Interactions Between Mast Cells and B Cells

**Figure 1:**
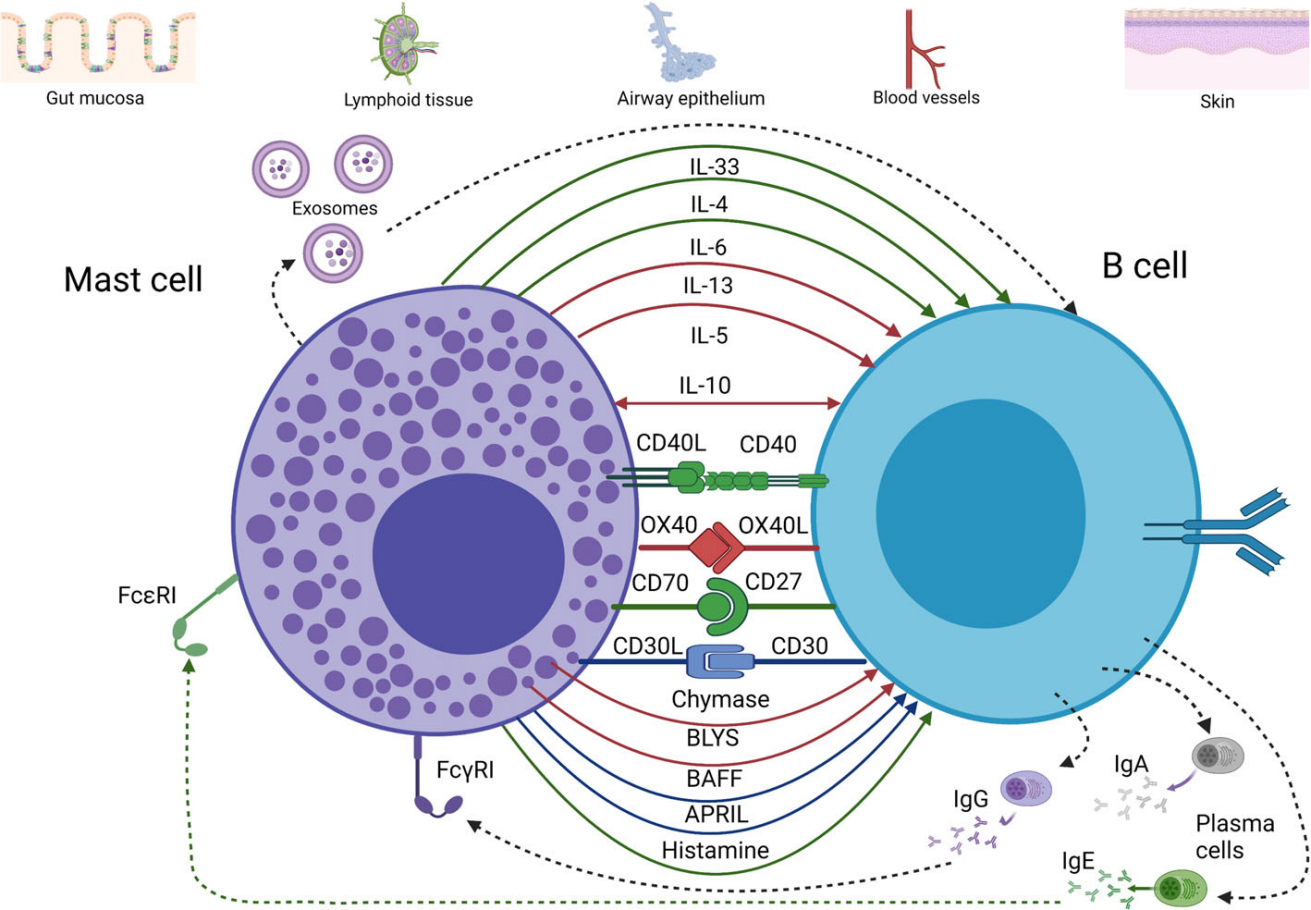
Major pathways of communication between mast cells and B cells. Evidence of cytokine and receptor-ligand interaction between mast cells and B cells has been depicted according to the following color scheme: red for evidence found in rodents, blue for evidence found in humans, and green for rodents and humans. Interaction between mast cells and B cells can occur at mucosal sites as well as at lymphoid and vascular tissues (although less frequently than at the mucosa). This is achieved by a broad array of cytokines (mainly type 2 cytokines, IL-10, IL-6, and IL-33), membrane-bound receptors and ligands (e.g., CD40/CD40L), and granule products such as histamine and proteases. These interactions can promote B cell proliferation, survival, class-switch to IgA or IgE, among other impacts. In addition, exosomes from both mast cells and B cells may be involved in communication between these cells. (*Figure was prepared using BioRender*).

The potential and proven interactions between mast cells and B cells are complex and multifaceted. In considering these, it is important to distinguish between evidence obtained from human studies and those observed in rodent models. The use of mast cell lines without confirmation using primary mast cells in some studies also means that findings need to be interpreted with caution. Interactions between mast cells and B cells are summarised in **Figure 1**, including the important cell contact-dependent and mediator-dependent interactions.

### CD40/CD40L

The CD40/CD40L interaction is pivotal to the regulation of antigen presentation, T cell-dependent class-switching, memory B cell development, and germinal centre formation (28). The early recognition that mast cells express CD40L suggested additional roles for mast cells in modifying B cell functions. This included the demonstration that mast cells can promote B cell class-switch to IgE production *via* a CD40/CD40L-dependent mechanism in the presence of IL-4 (29).

Signalling through CD40 was also shown to increase B cell proliferation by physical cellular contact (30). CD40L-expressing mast cells can enhance CD40/CD40L communication by promoting CD40-expression on B cells (30). Moreover, CD40L can be upregulated on mast cells through the actions of invariant NKT (iNKT) cells. iNKT cells recognise CD1d on the surface of murine mast cells and trigger the upregulation of CD40L, which can subsequently stimulate IgE class-switch by B cells to enhance allergic airways responses (31).

The CD40/CD40L-axis seems to play a role in local immunosuppression and immune tolerance, as it is implicated in the generation of IL-10 secreting B cells, as shown by Mion et al. (32). Indeed, the presence of mast cells enhances the development of B cells capable of producing IL-10 when appropriately stimulated, known as “IL-10 competent B cells”. Mast cells do not selectively enhance IL-10 production, on a per cell basis, but have a key role in enhancing expansion of regulatory B cell (Breg) subsets producing this anti-inflammatory mediator (32). Breg cell generation could be enhanced without direct cell contact, as exosomes from mast cells contain CD40L.

The ability of mast cells to enhance Breg development *via* a CD40-dependent mechanism also appears to be dependent on the anatomical site or other microenvironmental factors. In mice, the presence of mast cells does not enhance Breg differentiation in the spleen or peritoneal cavity but is important in the colon (32). This may be related to the presence of microbial factors in the intestine that are known to activate mast cells (33–35). This type of mast cell-B cell cross-talk may be more important in reducing local inflammatory responses at sites of microbial challenge and ensuring appropriate responses to damage or infection. In the context of allergic disease, mast cells and B cells have also been shown to co-localise in the airway epithelium of ovalbumin (OVA) challenged mice. Both CD40 and CD40L expression were upregulated in this setting possibly due to upregulation of Transglutaminase 2 triggered by antibody-antigen stimulation of mast cells (36). CD40 has also been shown to be expressed by airway epithelial cells (37), where it has been implicated in promoting T cell activation. These observations suggest that blockade of CD40/CD40L interactions in the allergic airways might have multiple consequences for local immune and inflammatory regulation. Notably, mast cells do not appear to have a key role in the development of mucosal tolerance, at least in adult animals (38).

### OX40/OX40L

Human mast cells from several tissues including the airways express OX40L (39), and this has been shown to provide a mechanism whereby mast cells may promote T cell responses. As mentioned previously, Hong et al. (40) have demonstrated that mast cells and B cells co-localise in the lung epithelium of OVA-sensitised mice. Moreover, expression levels of OX40/OX40L and CD40/CD40L were elevated. Inhibition of these pathways decreases the levels of OVA-specific IgA and IgE and reduces antigen-dependent mediator-release by mast cells. This model shows how B cells are activated by mast cells through the CD40/CD40L pathway as well as the OX40/OX40L-axis, in the presence of appropriate cytokines such as IL-4, IL-13, IL-6, and TGF-β. These signal through the TRAF2-MEKK1 and TRAF60-TAK1 signalling pathways, respectively, to induce B cell class-switch into IgA and IgE secreting cells. Enhanced IgE could provide positive feedback by stimulation of FcϵRI in mast cells, which in turn would increase mediator production and release. In addition to these more direct interactions between mast cells and B cells through OX40-dependent mechanisms there are a host of impacts that may result from mast cell-T cell interactions through either cell-cell contact or exosomes. For example, it has been clearly demonstrated that mast cells can limit the actions of regulatory T cells (Tregs) and promote Th17 production *via* an OX40/OX40L and IL-6-dependent mechanism (41).

### CD30/CD30L

Mast cells can express both CD30 and CD30L. CD30 expression on mast cells is often associated with mastocytosis or chronic inflammation. In contrast, CD30L expression by mast cells is more consistently observed in a variety of tumours and tumour-draining lymph nodes (42). Molin et al. ^74^ demonstrated that human mast cells interact with Reed-Sternberg lymphoma cells through the CD30/CD30L axis in Hodgkin’s Lymphoma, leading to an increase in proliferation of the latter. It has also been shown that CD30L-signalling induces mast cells to produce chemokines such as CXCL8 without evidence of degranulation or lipid mediator production (43). However, the ability of this interaction to induce chemokines that would induce B cell migration has not been directly examined. Only very specific subsets of B cells in the germinal centre and extrafollicular environment normally express CD30. Notably, the human CD30+ extrafollicular B cells are a subset of active memory B cells (44). It is plausible that mast cells also interact with these B cell populations to promote proliferation under some circumstances.

### CD27 and CD52

Early mast cell progenitors have been described as CD27+ (45) and mast cells ex vivo have also been described to express CD70. This was particularly studied in patients with Waldenström macroglobulinemia, a form of lymphoplasmacytic lymphoma. In this setting, soluble CD27 produced by lymphoplasmacytic cells upregulated CD40L on mast cells (30). CD40/CD40L interactions can promote the proliferation of malignant cells and have therefore been implicated as a negative factor in disease progression. It is plausible that this mechanism may extend outside of malignancy, perhaps to a subset of antibody-producing cells.

Both mast cells and B cells express the 12-amino acid GPI linked peptide CD52 (46), which is thought to have a role in retaining cell mobility. CD52 can ligate with Siglec-10 found on mast cells in addition to sialic acid. However, the precise role of this interaction is unknown. Siglec-10-signalling *via* CD24 can reduce responses to DAMPs and inhibit responses to tissue injury in humans (47). This may provide a mechanism whereby local inhibition of inflammation may result from mast cell-B cell cross-talk. However, given the wide range of cell types expressing these molecules it may be a more general method to regulate immune responses in certain tissues.

## Mast Cell Mediator Impacts on B Cells

In response to a variety of stimuli including pathogen-associated molecular patterns (PAMPs) or damage-associated patterns (DAMPs), mast cells selectively produce a subset of their wide array of potential mediators. These include preformed granule-associated products such as proteases and histamine, newly formed lipid mediators such as LTC_4_ and PGD_2_, and over 40 different cytokines and chemokines. The combinations, timing and range of such mediator production is dependent on the nature of the acute stimulation, the microenvironment, and the mast cell subpopulation. Many mast cell mediators can directly or indirectly modify B cell recruitment, function, or differentiation. The following section will outline mediators important in the setting of acute or chronic infection.

### Histamine

Histamine is a biogenic amine released by mast cell granules during allergic reactions and in response to multiple other stimuli that induce mast cell degranulation such as tissue injury, and responses to certain pathogen products. The impact of histamine on immunity has been extensively studied as reviewed by Akdis and Blaser (48). In mice, deletion of H1R resulted in suppression of IFN-γ and enhanced secretion of the type 2 cytokines, IL-4 and IL-13, with subsequent impacts on B cell responses (49). B cells express both H1 and H2 receptors that impact cellular functions. Early reports demonstrated that H1-signalling with IgM/antigen-stimulation enhanced splenic B cell proliferation (50). Kimata et al. (51) showed that B cells from healthy donors treated with anti-CD58 plus IL-4 or IL-13 enhanced IL-6 and IL-10 production when concurrently treated with histamine. This in turn, selectively increased IgE and IgG4 secretion. However, as reviewed by Merluzzi et al. (52) the impact of adding histamine in B cell culture systems has reportedly been variable, possibly due to its short half-life and the presence of histamine degrading enzymes.

H2 receptors on human cells including B cells are endogenously active, so while histamine can enhance their activity, important clues to their regulatory function can often be best obtained by using H2 antagonists or through studies of receptor-deficient cells or animals. Notably, the widely used H2 antagonists, ranitidine and famotidine have been shown to have some significant impacts on B cell activity. For example, ranitidine reduced tumor growth *via* a B cell-dependent mechanism in murine models of breast cancer (53). More recently, Meghnem et al. (54) showed that high dose ranitidine inhibited the number of circulating CD19+ B cells in 29 healthy human subjects. It is not known if chronic histamine stimulation in the context of allergic disease enhances such B cell populations.

### Proteases

Mast cell proteases are released in large amounts from activated mature and immature mast cells, particularly during degranulation. They have a wide variety of important functions in aiding host defence and enhancing the function of numerous cytokines through protease-mediated activation during infection, especially at mucosal surfaces. These include TGF-β family members that play key roles in regulating B cell development and in promoting IgA class-switch essential for the appropriate immune protection of mucosal surfaces [reviewed in (55)] as well as impacts on the activity of other inflammatory cytokines such as IL-33 (56–58). Chymase enzymes from mast cells have also been shown to induce B cell secretion of IgG1 and IgE as shown by Yoshikawa et al. (59) using rat mast cell protease II, although the mechanism for this is unclear.

Tryptase production by mast cells can also influence local mediator production and the cellular microenvironment. Moreover, tryptase can activate several protease-activated receptors such as protease activated receptor 2 (PAR2). Xue et al. (60) showed that B cells constitutively express PAR2, with levels increasing in allergic rhinitis. After PAR-2 activation, signalling through Bcl2L12 leads to IL-10 transcription repression and reduced IL-10-expression by B cells from patients with allergic rhinitis. The fact that tryptase is an important activator of PAR2 adds to the important role of mast cells in an allergic setting and raises the possibility of reduced tolerogenic responses following mast cell degranulation through inhibition of IL-10 production by B cells. It remains to be discovered if this mechanism occurs in other tissue settings where mast cells and B cells co-localise such as the gut.

### IL-6

Mast cells can be a rich source of IL-6 in response to certain infections. For example, when activated with high doses of *Escherichia coli* lipopolysaccharides (LPS), rodent mast cells have been reported to produce more IL-6 on a per cell basis than similarly treated macrophages. IL-6 has several roles in regulating B cell and plasma cell development and was originally described as a B cell differentiation factor. IL-6 is crucial for development of immunoglobulin-producing plasma cells and in some cases, class-switching. Merluzzi et al. (30) have shown that mast cells promote B cells differentiation into plasma cells with an IgA isotype through IL-6 secretion, which suggests that B cells can class-switch to IgA without T cell help. This process may be particularly important in the context of mucosal infections and host defence in airways. Another IL-6 family member, leukemia inhibitory factor, produced by mast cells (61), has been shown to selectively activate B1a cells in mice (62). It has also been suggested that in the presence of microbial stimulation IL-6 can promote the generation of Bregs (63). This adds to the complexity of the potential impact of mast cell IL-6 production following microbial breach of the epithelial barrier or mast cell contact with bacterial products.

Mast cells can also impact tissue remodelling events through IL-6. As described by Breitling et al. (64) in a murine pulmonary hypertension model, mast cells promoted vascular remodelling of the pulmonary artery of rats. A genetic analysis of lung samples from these rats revealed increased immunoglobulin gene products relative to controls, indicating a link between mast cells and immunoglobulin production. Upon further investigation, mast cell-derived IL-6 proved important. Pulmonary hypertension was improved by IL-6 inhibition, although increased mast cell density persisted when IL-6 was diminished. The depletion of B cells with anti-CD20 as well as the use of B cell-deficient mice also improved pulmonary hypertension. Along those lines, autoantibody levels as well as vascular remodeling decreased after oral ketotifen treatment, a mast cell stabiliser. Further information on mast cell/B cell communication will be crucial for devising novel strategies to treat pulmonary hypertension.

### Interferons and B Cell Chemoattractants

Mast cells are not thought to be a significant source of type 2 IFNs (e.g., IFN-*γ*), although they can produce it under some circumstances (65). Mast cells are, however, an important source of type 1 IFNs following viral infection (11, 12, 18). The IFN response of human mast cells to viral infection can be enhanced by IL-4 (66) with potential to modify B cell activity. It has been demonstrated that type 1 IFN-signalling in B cells can lead to the loss of tolerance and the development of autoreactive B cells (67). Type 1 IFN responses by mast cells are only one of a number of possible routes by which they may enhance antibody generation in autoimmune disease, such as their enhancement of anti-citrullinated protein antibodies in rheumatoid arthritis (68). This role for IFNs is best studied in the context of systemic lupus but could potentially also enhance responses to environmental allergens at sites such as the nasal mucosa or intestine which are prone to viral infection.

Human mast cells are also an important source of type 3 IFNs such as IL-29 during select viral infections and upon activation by specific viral-associated products such as double stranded RNA. IFN-λ has been demonstrated to enhance the differentiation of naïve B cells into plasmablasts *via* the mTORC1 pathway (69). While the contribution of mast cells to this and other IFN-dependent modulation activities on B cells is likely small within classical draining lymph node sites, it may be of greater importance at sites of mast cell-B cell co-localisation and viral exposure, such as the nasal mucosa, intestinal lamina propria, or inflamed skin.

In environments with high levels of IFNs such as sites of viral infections, mast cells can produce large amounts of several lymphocyte chemoattractants (70). Indeed, mast cells can produce CXCL10 in the context of reovirus, dengue virus, influenza, and RSV infection (71). CXCL10 has B cell and T cell chemoattractant abilities in addition to multiple other impacts. CXCL13 is also recognised as a critical B cell chemoattractant and although not widely studied from mast cells has also been reported to be produced following reovirus infection of human cord blood-derived mast cells (13).

### IL-33 and Type 2 Cytokines

Mast cells can be a significant source of type 2 cytokines that impact B cell development and function. For example, the production of IL-4 by mucosal mast cells in allergic rhinitis may enhance IgE class-switch by B cells, albeit to a lesser degree than IL-13 (72). Local microbial stimulation likely contributes to such mast cell activation at mucosal settings. IL-33, a member of the IL-1 receptor family, has also been reported to strongly influence B cells. IL-33 has been reported to induce activation of murine B1 cells through the ST2 receptor and drive the production of CCL2 and CCL3, chemokines involved in the trafficking of monocytes, macrophages, and other effector cells. Stimulation of B1 cells with IL-33 also generates the production of angiogenic factors such as VEGF and GM-CSF (73). IL-33 also stimulates IL-5 production and secretion in both mast cells and B cells, which leads to paracrine and autocrine stimulation through the IL-5 receptor to promote B cell proliferation, maintenance of Bregs, and antibody production (52). Overall, mast cells both produce IL-33 and respond to this alarmin through the production of similar mediators. Therefore, it possible that mast cell/B cell interaction at mucosal sites can perpetuate local inflammatory responses in specific settings through production of cytokines and angiogenic factors.

### BAFF and APRIL

Mast cells not only influence B cells in terms of class-switching and differentiation but also produce soluble mediators from the TNF ligand family that enhance B cell survival and limit apoptosis. As shown by Wang et al. (74), among many cell types, mast cells produce B cell activating factor (BAFF) in the ectopic lymphoid tissue of nasal polyps. Increased BAFF production may promote B cell survival to potentially promote ectopic lymphoid tissue formation. In support of this, another related member of the TNF ligand family, A Proliferation-Inducing Ligand (APRIL) along with B-Lymphocyte Stimulator factor (BLYS) can promote the survival of lymphoplasmacytic cells in Waldenström macroglobulinemia (75). Mast cells produced APRIL in response to CD70 stimulation through CD27 (75). The activities of these molecules, together with CD40/CD40L interactions support the importance of a range of TNF family members in mast cell/B cell interaction.

### Exosomes

Mast cells may also modulate B cells through transfer of exosomes. Mast cell-derived exosomes can harbour multiple molecules highlighted herein (e.g., CD40 and CD40L) and others such as CD86, MHC II, LFA-1, and ICAM-1 (76). Exosome secretion may be dependent on IL-4 and mast cell maturity. Paradoxically, exosomes from mast cells have been shown to induce secretion of IL-2, IFN-γ and IL-12, skewing the immune response to type 1 cytokine polarisation. This indicates that mast cells can broadly shape immune responses (e.g., allergic reactions) including the intensity through their exosome contents. Despite strong evidence of exosome importance for modifying B and T cell activities *in vitro* (77–79), the role of mast cell exosomes has yet to be conclusively demonstrated *in vivo*, particularly in settings of infection.

## Mast Cell/B Cell Interaction and Co-Localisation

**Figure 2:**
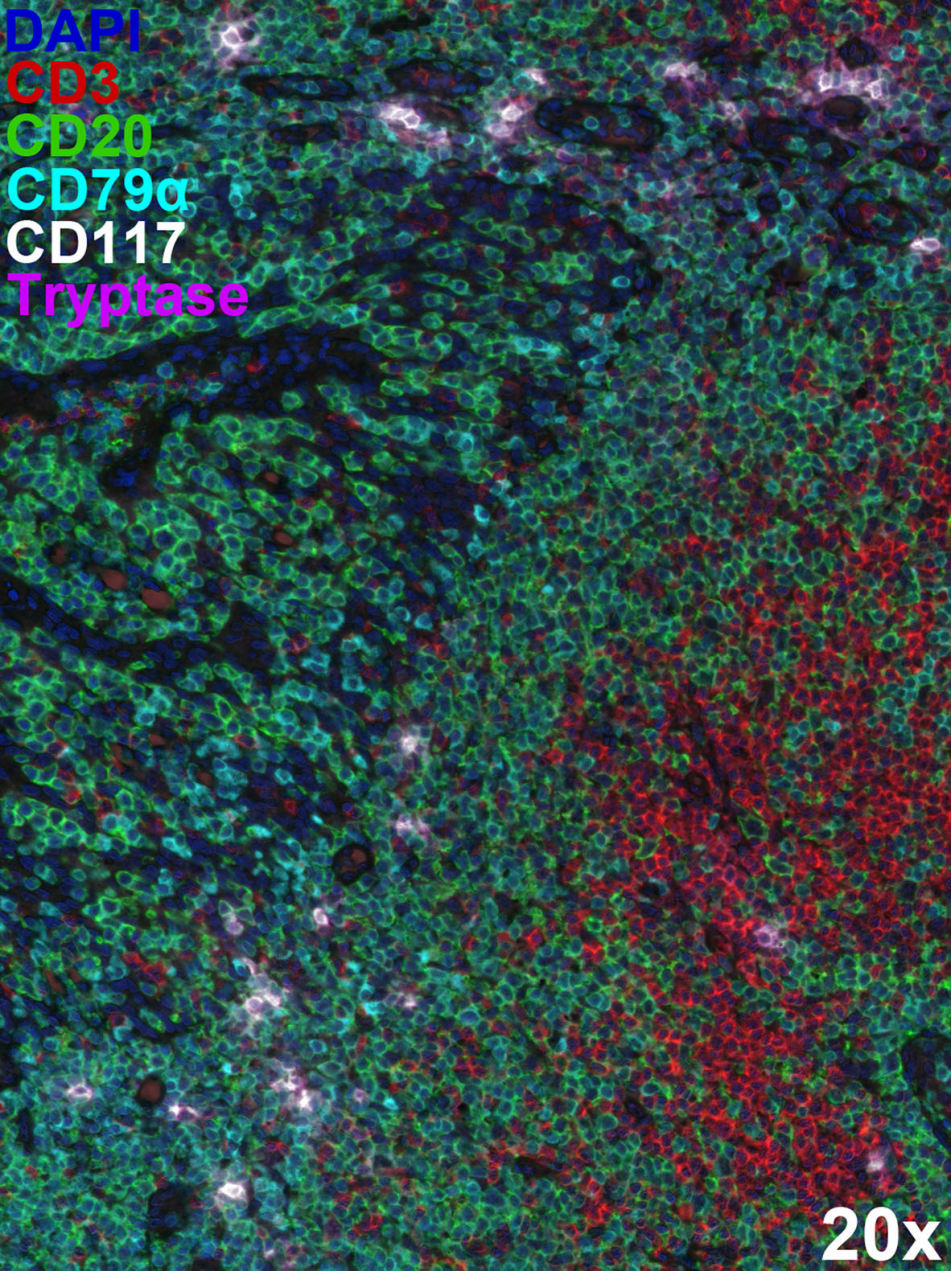
Extensive mast cell/B cell co-localisation within lymphoid tissue. Multiplex immunostaining using Opal™ technology (Akoya Biosciences) identified CD20^+^CD79α^+^ B cells (green and cyan), tryptase^+^CD117^+^ mast cells (magenta and white), and CD20^-^CD3^+^ T cells (red) within formalin-fixed, paraffin-embedded 5 µm-thick human tonsil sections. The 6-plex panel (including DAPI counterstaining) scans were acquired on the Mantra 2™ Quantitative Pathology Workstation using pre-defined parameters at 20x original magnification and spectrally unmixed using inForm^®^ software (Akoya Biosciences). Representative image illustrating close-proximity between mast cells and B cells within tonsil architecture. Mast cells appear less in T cell dense areas.

Mast cells are resident tissue cells observed in high density at mucosal surfaces and in the skin. However, they are also found throughout the body, mainly in association with blood vessels. Mast cells are present in lymphoid tissue, but usually not as a major resident population. However, mast cell migration to inguinal lymph nodes has been reported in the context of early inflammatory responses (e.g., following UV exposure) (80–82). Mast cells are also found in close-proximity to B cells in tonsil and Peyer’s patches (81–83) (**Figure 2**). At sites of ectopic lymphoid tissue development, such as in the airways during chronic allergic disease (84) and in the joints during rheumatoid arthritis (68, 85), mast cells and B cells are also found within the same microenvironment at high density, potentially as a result of the disease (**Table 1**).

**Table 1:** Sites of close-proximity between mast cells and B cells in multiple hosts.

Sites of close-proximity	Mast cell identifier(s)	B cell identifier(s)	Host	Citation(s)
Gut mucosa	Tryptase^+^	CD20^+^	Human	Merluzzi et al., 2010 (30)
Airway epithelia	CD117 (c-Kit)^+^	CD23^+^	Mouse	Hong et al., 2013 (36)
Lungs	Tryptase^+^	CD45RA^+^	Rat	Breitling et al., 2017 (64)
Inguinal lymph node	Toluidine blue^+^	CD19^+^	Mouse	Byrne et al., 2008 (80)
	Tryptase^+^	CD20^+^	Human	Merluzzi et al., 2010 (30)
Tonsil	CD117^+^	CD20^+^	Human	Rivellese et al., 2018 (68)
	Alcian blue^+^		Human	He and Xie 2005; He et al., 2005 (81, 82)
Ectopic lymphoid tissue	Tryptase^+^	CD19^+^	Human	Zhai et al., 2018 (84)
	CD117^+^	CD20^+^	Human	Rivellese et al., 2018 (68)

This co-localisation may be functionally important. Early studies demonstrated that mast cells could effectively promote B cell function and proliferation (86, 87). There is evidence for interaction between mast cells and B cells at the nasal mucosa of patients with allergic rhinitis. Mast cells have been shown to induce IgE synthesis by purified tonsillar B cells in response to antigens without the need for exogenous IL-4 or IL-13. Mast cells in this context were reported to have enhanced expression of FcϵR1, CD40L, IL-4, and IL-13 (72). *In vitro*, Palm et al. (88) demonstrated in mice that degranulated (and to some extent resting) mast cells enhanced B cell activation including elevated expression of CD19, MHC II, CD86, and L-selectin. Such activated B cells also secreted greater amounts of IgG and IgM. In the intestine, a different impact of mast cell-B cell interaction has been suggested. Mast cells and B cells co-localise in the lamina propria of the intestine of individuals with inflammatory bowel disease and an association has been reported between the presence of mast cells and elevated IgA secretion (30). These authors also clearly demonstrated the presence of mast cells and B cells in human lymph nodes undergoing reactive hyperplasia. Mast cells were particularly localised surrounding follicles and in the paracortical and medullary regions of the lymph nodes, raising the possibility of interaction with recirculating naïve B cells and activated post germinal centre B cells. Using a co-culture of primary mast cells and B cells, the presence of mast cells promoted the survival and proliferation of B cells through an IL-6-dependent mechanism which also required cell-cell interactions such as CD40/CD40L (30). In contrast to these findings, it has also been suggested that mast cells within lymph nodes may have more suppressive immunomodulatory functions which limit B cell responses. For example, Chacon Salinas et al. (89) have demonstrated that the production of IL-10 by mast cells can indirectly disrupt germinal centre formation *via* impacts on T follicular helper cells. The production of IL-10 and IgA by B cells has also been implicated in reducing neuroinflammation (90) and inflammatory responses at mucosal surfaces (91). Thus, any impact of mast cells in modifying such responses could have critical downstream impacts on the mucosal immune environment.

Outside of secondary and ectopic lymphoid tissues there are other environments where mast cells and B cells may be concentrated. These include the previously mentioned lamina propria of the intestine as well as several tumour settings. Bone marrow studies in patients with Waldenström macroglobulinemia have shown an increased number of mast cells, where they are thought to contribute to tumour growth as well as angiogenesis, as shown by Ahn et al. (92). In addition to an increase in mast cell density, the proportion of CD40L+ mast cells correlated with poor tumour prognosis, illustrating the potential cross-talk between B cells (albeit abnormal ones) and mast cells through the CD40/CD40L axis. A similar link has been suggested in the setting of multiple myeloma as shown by Pappa et al. (93). Neoplastic B cells may also modify the activities of mast cells such as recruitment and activation. This is in addition to a multitude of less B cell specific interactions between mast cells and tumours that occur in tumour settings (94–97). For example, Fischer et al. (98) have shown that neoplastic cells from Hodgkin’s Lymphoma secrete CCL5 at sites of infiltration to recruit mast cells. Mast cells may also impact other aspects of the tumour microenvironment. For example, increased numbers of mast cells are found in nodular sclerosing forms of Hodgkin’s lymphoma, associated with tissue fibrosis and the presence of IL-13 expressing Reed Sternberg cells. Moreover, mast cells were associated with greater disease progression and increased micro-vessel density in primary cutaneous B cell lymphomas and have potential as a prognostic marker (99).

## Mast Cells and Chronic Infection

### Parasite Infections

Mast cells have important roles in many globally important parasite infections, although their contribution differs with the infecting microorganism. Helminth gastrointestinal infections are associated with increased mast cell density and activation at the site of infection, which contributes to epithelial sloughing and increased motility (100–102). Mast cells may also contribute to tissue repair through impacts on fibroblast activation and tissue remodeling. Mast cell proteases are directly toxic to many helminths (103) and the granule product chondroitin-sulphate has also been reported to be active (104) in preventing nematode adhesion and penetration of the intestinal mucosa. Helminth infection-associated mast cell activation likely also modifies the responses of local B cells and plasma cells through mediator impacts, some of which are detailed below.

Mast cells participate in shaping type 2 immune responses to many nematodes through the release of IL-33, IL-25, and thymic stromal lymphopoietin. As shown also by Hepworth et al. (100, 105, 106), mast cell-deficient mice had an impaired type 2 immune response against helminths. Enhanced type 2 immune responses could indirectly alter B cell responses and contribute to the high IgE levels observed in many such infections. In secondary infections, the presence of specific IgE may enhance local mast cell responses enabling more rapid infection clearance (100, 106).

Infections by *Schistosoma mansoni*, a parasite mainly found in Africa and South America, are known to induce polyclonal B cell responses. *S. mansoni* produces glycoproteins recognised by galectin-3 that induce strong antibody responses. In a chronic schistosomiasis murine model developed by Oliveira et al. (107), mast cell degranulation was suggested to be one of the drivers for IgA class-switch and subsequent antibody production by peritoneal B1 cells. This process was regulated by galectin-3. Even though these findings are not yet completely understood, they highlight complex interactions between mast cells and B cells in parasite infections.

It is important to stress that many mast cell effector functions in response to chronic or repeated infection require the presence of B cells and antibodies. This was formally demonstrated by Matsumoto et al. (108) using activation induced cytidine deaminase (AID)-deficient mice, which despite the presence of intact T and mast cell compartments demonstrated delayed elimination of *Strongyloides venezuelensis*. Enhanced expulsion was restored with the addition of IgG1 and IgE to AID-deficient mice indicating the importance of antibody production. Antibody enhanced expulsion was shown to be mediated by mast cells, which underscores the importance of mast cell stimulation through their Fcγ and Fcϵ receptors (108).

While the role of mast cells and B cells in helminth infections is well established, their role in protozoan infections is less clear. In infections by *Plasmodium* spp., mast cell-derived TNF-α was shown to be important for the clearance of infection and protection against cerebral involvement. However, mast cell degranulation, increased concentrations of histamine and higher levels of IgE against *Plasmodium falciparum* were associated with worse outcomes. Similarly, in the setting of cerebral malaria, mast cell degranulation involved histamine release, increased vascular permeability, enhanced endothelial damage, and lead to the release of VEGF, which associated with worse outcome (109).

In infections by *Leishmania* spp., the participation of mast cells seems to depend on the species of the parasite as a first line of defence, without a clear interaction with B cells. As shown by Naqvi et al. (110), mast cells can phagocytose *L. tropica* which causes cutaneous leishmaniasis but not *L. donovani*, which causes visceral leishmaniasis. Both species are susceptible to killing by extracellular traps created by mast cells, but *L. tropica* is more vulnerable. These observations highlight differential roles for mast cells depending on the type of leishmaniasis.

In Chagas disease, there have been descriptions of co-existence of mast cells and B cells in heart biopsies of patients with dilated myocardiopathy. The presence of mast cells in the heart and intestine has been associated with worse prognosis, possibly implying ongoing inflammation and fibrotic processes involving mast cells (111–113).

### Viral Infections

Mast cells have been implicated in the host response to multiple viral infections. Multiple viral associated stimuli can induce the production of type I and III IFNs, chemokines, inflammatory cytokines as well as factors involved in tissue remodelling such as VEGF (11, 17, 70). Such interactions occur in the absence of B cells or antibody in many cases. For example, infections with reovirus, respiratory syncytial virus, adenovirus, and influenza of human mast cells have been reported, leading to substantial mediator release (11, 13, 16, 114–116). These types of responses are often the result of signalling through RNA or DNA sensors such as TLR 3, 7, 8 or 9, RIG-I, MDA-5, and STING pathways (12, 117, 118). Mediators produced can include both B cell chemoattractants and cytokines which act on B cells, such as IL-6. However, antibody mediated events can also play a key role in these processes for some viruses.

Antibody-dependent enhancement occurs when sub-neutralising levels of antibody facilitate the entry of virus into the cell *via* Fc receptors. For mast cells this process has been described for Dengue virus infection, mediated by low concentrations of IgG *via* Fc*γ*RII (15, 119) where it can induce cytokine and chemokine production, and mast cell apoptosis (120). IL-1β and TNF-α which have been implicated in vascular damage and endothelial dysfunction (121) are also produced by infected human mast cells. Mast cell activation in this clinical context has been implicated in Dengue Hemorrhagic Fever and Dengue Shock Syndrome (122–124).

Antibody-dependent enhancement of infection of mast cells may also be a factor in response to subsequent infections with other viruses such as Zika virus (116) and has most recently been suggested as a factor in SARS-CoV-2 infection (125, 126). It likely also occurs for other viruses under specific circumstances where antibody is not sufficient for neutralisation.

Antibody-mediated processes in viral infections can also activate mast cells *via* Fc receptor cross-linking leading to degranulation, lipid mediator production, and subsequent longer-term cytokine and chemokine production. The nature of the responses is highly dependent on the class and subclass profile of antibodies produced in response to infection. In many cases the type 1 cytokine response to viruses does not support a strong specific IgE response, but given the long half-life of such antibodies on mast cells in sites such as the skin and airways there is potential for IgE mediated events to enhance events such as the mobilisation of dendritic cells (24, 26) leading to enhanced B and T cell responses in draining lymph nodes. During HIV infections, mast cells and their precursors may also harbour virus which can be reactivated through either TLR- or antibody/Fc receptor-pathways (114, 127, 128). Indirect processes such as complement product (C3a, C5a) mediated activation of mast cells may also occur in response to antibody complexes with viral products.

### Atopy and Bacterial Infections

Mast cells and B cells are both important players in effective responses against bacterial pathogens. Mast cells function as critical sentinel cells during the early stages of infection and promote the recruitment of effector cells to sites of infection. On the other hand, the B cell mediated antibody response is key to combatting longer-term and secondary infections. Interplay between these cell types can therefore generally be seen as positive for anti-bacterial host defence. However, this is not always the case. The skin of individuals with atopic dermatitis is frequently colonised by *Staphylococcus aureus*. This suggests that *S*. *aureus* may contribute to the pathophysiological events that culminates in atopic dermatitis. Interestingly, mast cells and B cells may partner in driving this disease. A report using an atopic dermatitis mouse model showed that mast cell-deficient mice inoculated with wild-type *S*. *aureus* and challenged with ovalbumin had reduced skin disease and serum IgE than wild-type mice. Mast cell contribution may be through their degranulation products, as skin-derived murine mast cells degranulate in response to *S*. *aureus*-derived δ-toxin, which is enhanced if mast cells are first primed with B cell-derived IgE (129). Other Gram-positive bacterial-derived products promote mast cell production of type 2 cytokines known to induce atopic dermatitis features such as IL-13, as well as proinflammatory TNF-α and IL-6. Patients with atopic dermatitis have increased blood IgE production signatures in blood and higher frequency of blood and lesional B cells compared to control groups (130) but the precise contribution of mast cells to this response is unknown.

In related studies considering superantigens as triggers for atopic disease, Schlievert et al. (131) analysed the response of keratinocytes to bacterial superantigens and found an enhanced production of chemokines and cytokines. Among them, IL-33 was notably increased. Mast cells can also respond to IL-33 producing a number of type 2 cytokines which could promote class-switch to IgE and antibody generation. Taken together, these studies indicate an interplay between staphylococci colonisation, superantigen stimulation, mast cell stimulation and therefore B cell stimulation, and production of IgE which in turn could produce a positive feedback on mast cell degranulation.

### Hypersensitivity and Fungal Infections

Interaction between B cell and mast cells could also be an influencing factor in immunity and inflammation in response to several fungal infections. Histamine release from mast cells is a frequent feature of cutaneous and mucosal fungal infections. Allergic bronchopulmonary aspergillosis (ABPA) has been mainly reported in patients with asthma and cystic fibrosis where the production of IgE towards *Aspergillus* spp. spores by B cells leads to the activation of mast cells (132). *A. fumigatus* antigen Af1 is presented through MHC II to Th2 lymphocytes (132) eliciting production of IL-4 and IL-13. This type 2 cytokine rich environment promotes class-switch to IgE. As with other pathogens, IgE bound to mast cells can mediate mast cell activation to *Aspergillu*s spp. antigens in secondary or chronic infections (133). It is also worth noting that *Aspergillus* spp. can induce mast cell degranulation independently of IgE in rodents, without causing any damage to the hyphae (134) but more damage to the airway mucosa.

While the fungus itself produces damage in the airway epithelium, the release of mast cell proteases and the recruitment of eosinophils contribute substantially to remodelling of the airway in response to such infection (135). Eosinophils release their toxic granular proteins while mast cells release tryptase and both of them activate and promote the production of TGF-β. This cytokine induces bronchial fibroblasts to differentiate to myofibroblasts that directly induce in the remodelling of the airway wall (135). Further corroborating the involvement of mast cells driven by IgE in ABPA, treatment with omalizumab (a monoclonal antibody targeting the high-affinity receptor binding site on human IgE and thereby reducing mast cell sensitisation) has been shown to be effective for patients with severe allergic asthma and ABPA (136).

Cross-talk between mast cells and B cells has also been shown to be important in *Malassezia* spp. infections. *Malassezia* spp. are a group of opportunistic fungi that grow mainly in skin areas with abundant sebaceous glands. They have been implicated in the pathogenesis of atopic eczema, seborrheic dermatitis and pityriasis versicolor (133). Selander et al. have shown, using a rodent model, that *Malassezia sympodialis* can activate both non-sensitised and IgE-sensitised mast cells (137). While IgE-sensitised mast cells degranulate, release cysteinyl leukotrienes, cytokines and chemokines when stimulated with extracts of *M. sympodialis*, non-sensitised mast cells selectively release leukotrienes without degranulation (137). This activation is induced through the TLR2/Myd88 and MAPK pathway. Although the *in vivo* impact of such responses is not well studied, cysteinyl leukotrienes can act through Cys LT1 receptors on B cells to enhance immunoglobulin production *in vitro* (138). Taken together, these findings support the idea of a mast cell response to *Malassezia* spp. enhanced by IgE produced by B cells in atopic dermatitis. This would be expected to both promote local inflammation and effector cell recruitment and potentially enhance the development and maintenance of an acquired immune response to infection through impacts on dendritic cells and draining lymph nodes.

Some similar mast cell responses have also been observed in response to *Candida albicans.* Mast cells can respond directly to this pathogen through both TLR and dectin-1 mediated pathways, giving rise to both lipid mediator and cytokine responses, often without degranulation. These responses may promote the generation of acquired immunity. A link has also been established between *Candida* spp. colonisation of the skin and exacerbation of atopic dermatitis (139, 140). As shown by various reports (139, 140), *Candida* spp. can induce IgE-mediated mast cell degranulation and subsequent responses in humans who have been previously sensitised and both promote inflammation and exacerbate histaminergic symptoms of patients. Taken together, these findings suggest a dual role of mast cells in the interaction with *Candida* spp.: they act as sentinels and first line of defence, but their mediators can become detrimental for the host and perpetuate inflammation.

## Conclusion

This short description of some of the most crucial known and potential interactions between B cells and mast cells raises many questions. Mast cells are tissue resident cells, often in limited numbers in lymph nodes. However, they are more prominent in tissues such as the tonsils and Peyer’s patches where they are in close-proximity to B cells as they are in the respiratory and intestinal mucosa. Traditionally, B cells in the skin have often been overlooked but both B1 and B2 cells are present and B cell populations in these sites are increased during inflammation. It is in the skin and other mucosal tissues where mast cells play the most important role as sentinel cells against infection; it is also in these sites where interaction between mast cells and B cells might be most critical to local infection and inflammation regulation. Given the key role of mast cells in promoting the selective recruitment and activation of other effector cells, it seems likely that mast cells play an early role in B cell responses. However, the complex interactions between mast cells and B cells persist longer-term. Current data suggests a number of mechanisms whereby mast cells can support or limit Breg development. Most of these can occur without direct cell contact. Similarly, the production of antibodies and cytokines such as IL-10 by the B cell lineage can dramatically alter mast cell function and provide a long-term mechanism for heightened responses to secondary infection. There is a wide menu of potential mechanisms whereby mast cells can modify B cell populations and function. Each tissue site and pathogen response will likely access only a subset of such mechanisms. Understanding and modifying these mast cell-dependent pathways shows promise for enhancing responses in chronic infection, limiting the development of autoreactive B cells and combating local immune suppression in some tumour settings. Critical to this process is direct analysis of the nature and function of local resident B cell populations and their resident mast cell neighbors in normal tissues and sites of disease.

## Author Contributions

AMP contributed the literature search, evaluation of material, **Figure 2** and a substantial portion of the written manuscript. MRH contributed **Figure 1**, evaluation of key sections of literature scientific insights and review. AMP contributed **Figure 1** and MRH contributed **Figure 2**. JSM provided substantial writing and editing of the manuscript and conceptualization of the review. All authors contributed to the article and approved the submitted version.

## Funding

This work was funded by the Canadian Institutes of Health Research (CIHR grant number PJT-173350). MH is the recipient of a Beatrice Hunter Cancer Research Institute trainee award. AP is funded through an investigator award from the IWK Health Centre, Halifax, NS.

## Conflict of Interest

The authors declare that the research was conducted in the absence of any commercial or financial relationships that could be construed as a potential conflict of interest.

## Publisher’s Note

All claims expressed in this article are solely those of the authors and do not necessarily represent those of their affiliated organizations, or those of the publisher, the editors and the reviewers. Any product that may be evaluated in this article, or claim that may be made by its manufacturer, is not guaranteed or endorsed by the publisher.

## References

[1] Mukai K Tsai M Saito H Galli SJ. Mast Cells as Sources of Cytokines, Chemokines, and Growth Factors. Immunol Rev (2018) 282(1):121–50. doi: 10.1111/imr.12634 PMC5813811 29431212

[2] Suto H Nakae S Kakurai M Sedgwick JD Tsai M Galli SJ. Mast Cell-Associated TNF Promotes Dendritic Cell Migration. J Immunol (2006) 176(7):4102–12. doi: 10.4049/jimmunol.176.7.4102 16547246

[3] Nakae S Suto H Kakurai M Sedgwick JD Tsai M Galli SJ. Mast Cells Enhance T Cell Activation: Importance of Mast Cell-Derived TNF. Proc Natl Acad Sci USA (2005) 102(18):6467–72. doi: 10.1073/pnas.0501912102 PMC1088381 15840716

[4] Arifuzzaman M Mobley YR Choi HW Bist P Salinas CA Brown ZD. MRGPR-Mediated Activation of Local Mast Cells Clears Cutaneous Bacterial Infection and Protects Against Reinfection. Sci Adv (2019) 5(1):eaav0216. doi: 10.1126/sciadv.aav0216 30613778 PMC6314830

[5] Malaviya R Ikeda T Ross E Abraham SN. Mast Cell Modulation of Neutrophil Influx and Bacterial Clearance at Sites of Infection Through TNF-Alpha. Nature (1996) 381(6577):77–80. doi: 10.1038/381077a0 8609993

[6] Marshall JS. Mast-Cell Responses to Pathogens. Nat Rev Immunol (2004) 4(10):787–99. doi: 10.1038/nri1460 15459670

[7] Dawicki W Jawdat DW Xu N Marshall JS. Mast Cells, Histamine, and IL-6 Regulate the Selective Influx of Dendritic Cell Subsets Into an Inflamed Lymph Node. J Immunol (2010) 184(4):2116–23. doi: 10.4049/jimmunol.0803894 20083654

[8] Leal-Berumen I Conlon P Marshall JS. IL-6 Production by Rat Peritoneal Mast Cells is Not Necessarily Preceded by Histamine Release and can be Induced by Bacterial Lipopolysaccharide. J Immunol (1994) 152(11):5468–76. 7514639

[9] Nakae S Suto H Iikura M Kakurai M Sedgwick JD Tsai M. Mast Cells Enhance T Cell Activation: Importance of Mast Cell Costimulatory Molecules and Secreted TNF. J Immunol (2006) 176(4):2238–48. doi: 10.4049/jimmunol.176.4.2238 16455980

[10] Dreskin SC Abraham SN. Production of TNF-Alpha by Murine Bone Marrow Derived Mast Cells Activated by the Bacterial Fimbrial Protein, FimH. Clin Immunol (1999) 90(3):420–4. doi: 10.1006/clim.1998.4657 10075872

[11] Al-Afif A Alyazidi R Oldford SA Huang YY King CA Marr N. Respiratory Syncytial Virus Infection of Primary Human Mast Cells Induces the Selective Production of Type I Interferons, CXCL10, and CCL4. J Allergy Clin Immunol (2015) 136(5):1346–54 e1. doi: 10.1016/j.jaci.2015.01.042 25819983

[12] Brown MG McAlpine SM Huang YY Haidl ID Al-Afif A Marshall JS. RNA Sensors Enable Human Mast Cell Anti-Viral Chemokine Production and IFN-Mediated Protection in Response to Antibody-Enhanced Dengue Virus Infection. PloS One (2012) 7(3):e34055. doi: 10.1371/journal.pone.0034055 22479521 PMC3316603

[13] Burke SM Issekutz TB Mohan K Lee PW Shmulevitz M Marshall JS. Human Mast Cell Activation With Virus-Associated Stimuli Leads to the Selective Chemotaxis of Natural Killer Cells by a CXCL8-Dependent Mechanism. Blood (2008) 111(12):5467–76. doi: 10.1182/blood-2007-10-118547 18424663

[14] Ebert S Becker M Lemmermann NA Buttner JK Michel A Taube C. Mast Cells Expedite Control of Pulmonary Murine Cytomegalovirus Infection by Enhancing the Recruitment of Protective CD8 T Cells to the Lungs. PloS Pathog (2014) 10(4):e1004100. doi: 10.1371/journal.ppat.1004100 24763809 PMC3999167

[15] King CA Anderson R Marshall JS. Dengue Virus Selectively Induces Human Mast Cell Chemokine Production. J Virol (2002) 76(16):8408–19. doi: 10.1128/JVI.76.16.8408-8419.2002 PMC155122 12134044

[16] Marcet CW St Laurent CD Moon TC Singh N Befus AD. Limited Replication of Influenza A Virus in Human Mast Cells. Immunol Res (2013) 56(1):32–43. doi: 10.1007/s12026-012-8377-4 23055084

[17] McAlpine SM Issekutz TB Marshall JS. Virus Stimulation of Human Mast Cells Results in the Recruitment of CD56(+) T Cells by a Mechanism Dependent on CCR5 Ligands. FASEB J (2012) 26(3):1280–9. doi: 10.1096/fj.11-188979 22125314

[18] Portales-Cervantes L Haidl ID Lee PW Marshall JS. Virus-Infected Human Mast Cells Enhance Natural Killer Cell Functions. J Innate Immun (2017) 9(1):94–108. doi: 10.1159/000450576 27806369 PMC6738812

[19] St John AL Rathore AP Raghavan B Ng ML Abraham SN. Contributions of Mast Cells and Vasoactive Products, Leukotrienes and Chymase, to Dengue Virus-Induced Vascular Leakage. Elife (2013) 2:e00481. doi: 10.7554/eLife.00481 23638300 PMC3639510

[20] Wang Z Lai Y Bernard JJ Macleod DT Cogen AL Moss B. Skin Mast Cells Protect Mice Against Vaccinia Virus by Triggering Mast Cell Receptor S1PR2 and Releasing Antimicrobial Peptides. J Immunol (2012) 188(1):345–57. doi: 10.4049/jimmunol.1101703 PMC3244574 22140255

[21] Iikura M Suto H Kajiwara N Oboki K Ohno T Okayama Y. IL-33 can Promote Survival, Adhesion and Cytokine Production in Human Mast Cells. Lab Invest (2007) 87(10):971–8. doi: 10.1038/labinvest.3700663 17700564

[22] Lunderius-Andersson C Enoksson M Nilsson G. Mast Cells Respond to Cell Injury Through the Recognition of IL-33. Front Immunol (2012) 3:82. doi: 10.3389/fimmu.2012.00082 22566963 PMC3342375

[23] Allakhverdi Z Smith DE Comeau MR Delespesse G. Cutting Edge: The ST2 Ligand IL-33 Potently Activates and Drives Maturation of Human Mast Cells. J Immunol (2007) 179(4):2051–4. doi: 10.4049/jimmunol.179.4.2051 17675461

[24] Shelburne CP Nakano H St John AL Chan C McLachlan JB Gunn MD. Mast Cells Augment Adaptive Immunity by Orchestrating Dendritic Cell Trafficking Through Infected Tissues. Cell Host Microbe (2009) 6(4):331–42. doi: 10.1016/j.chom.2009.09.004 PMC2764554 19837373

[25] Dudeck A Suender CA Kostka SL von Stebut E Maurer M. Mast Cells Promote Th1 and Th17 Responses by Modulating Dendritic Cell Maturation and Function. Eur J Immunol (2011) 41(7):1883–93. doi: 10.1002/eji.201040994 21491417

[26] Jawdat DM Albert EJ Rowden G Haidl ID Marshall JS. IgE-Mediated Mast Cell Activation Induces Langerhans Cell Migration *In Vivo* . J Immunol (2004) 173(8):5275–82. doi: 10.4049/jimmunol.173.8.5275 15470073

[27] Jawdat DM Rowden G Marshall JS. Mast Cells Have a Pivotal Role in TNF-Independent Lymph Node Hypertrophy and the Mobilization of Langerhans Cells in Response to Bacterial Peptidoglycan. J Immunol (2006) 177(3):1755–62. doi: 10.4049/jimmunol.177.3.1755 16849485

[28] Grewal IS Flavell RA. CD40 and CD154 in Cell-Mediated Immunity. Annu Rev Immunol (1998) 16:111–35. doi: 10.1146/annurev.immunol.16.1.111 9597126

[29] Gauchat JF Henchoz S Mazzei G Aubry JP Brunner T Blasey H. Induction of Human IgE Synthesis in B Cells by Mast Cells and Basophils. Nature (1993) 365(6444):340–3. doi: 10.1038/365340a0 7690905

[30] Merluzzi S Frossi B Gri G Parusso S Tripodo C Pucillo C. Mast Cells Enhance Proliferation of B Lymphocytes and Drive Their Differentiation Toward IgA-Secreting Plasma Cells. Blood (2010) 115(14):2810–7. doi: 10.1182/blood-2009-10-250126 20101023

[31] Hong GU Kim NG Kim TJ Ro JY. CD1d Expressed in Mast Cell Surface Enhances IgE Production in B Cells by Up-Regulating CD40L Expression and Mediator Release in Allergic Asthma in Mice. Cell Signal (2014) 26(5):1105–17. doi: 10.1016/j.cellsig.2014.01.029 24509414

[32] Mion F D'Inca F Danelli L Toffoletto B Guarnotta C Frossi B. Mast Cells Control the Expansion and Differentiation of IL-10-Competent B Cells. J Immunol (2014) 193(9):4568–79. doi: 10.4049/jimmunol.1302593 25267976

[33] Bednarska O Walter SA Casado-Bedmar M Strom M Salvo-Romero E Vicario M. Vasoactive Intestinal Polypeptide and Mast Cells Regulate Increased Passage of Colonic Bacteria in Patients With Irritable Bowel Syndrome. Gastroenterology (2017) 4):948–960 e3. doi: 10.1053/j.gastro.2017.06.051 PMC5623149 28711627

[34] Kramer S Sellge G Lorentz A Krueger D Schemann M Feilhauer K. Selective Activation of Human Intestinal Mast Cells by Escherichia Coli Hemolysin. J Immunol (2008) 181(2):1438–45. doi: 10.4049/jimmunol.181.2.1438 18606698

[35] Chichlowski M Westwood GS Abraham SN Hale LP. Role of Mast Cells in Inflammatory Bowel Disease and Inflammation-Associated Colorectal Neoplasia in IL-10-Deficient Mice. PloS One (2010) 5(8):e12220. doi: 10.1371/journal.pone.0012220 20808919 PMC2923184

[36] Hong GU Park BS Park JW Kim SY Ro JY. IgE Production in CD40/CD40L Cross-Talk of B and Mast Cells and Mediator Release *via* TGase 2 in Mouse Allergic Asthma. Cell Signal (2013) 25(6):1514–25. doi: 10.1016/j.cellsig.2013.03.010 23524335

[37] Propst SM Denson R Rothstein E Estell K Schwiebert LM. Proinflammatory and Th2-Derived Cytokines Modulate CD40-Mediated Expression of Inflammatory Mediators in Airway Epithelia: Implications for the Role of Epithelial CD40 in Airway Inflammation. J Immunol (2000) 165(4):2214–21. doi: 10.4049/jimmunol.165.4.2214 10925309

[38] Tunis MC Dawicki W Carson KR Wang J Marshall JS. Mast Cells and IgE Activation do Not Alter the Development of Oral Tolerance in a Murine Model. J Allergy Clin Immunol (2012) 130(3):705–715 e1. doi: 10.1016/j.jaci.2012.04.011 22607990

[39] Kashiwakura J Yokoi H Saito H Okayama Y. T Cell Proliferation by Direct Cross-Talk Between OX40 Ligand on Human Mast Cells and OX40 on Human T Cells: Comparison of Gene Expression Profiles Between Human Tonsillar and Lung-Cultured Mast Cells. J Immunol (2004) 173(8):5247–57. doi: 10.4049/jimmunol.173.8.5247 15470070

[40] Hong GU Lim JY Kim NG Shin JH Ro JY. IgE and IgA Produced by OX40-OX40L or CD40-CD40L Interaction in B Cells-Mast Cells Re-Activate FcepsilonRI or FcalphaRI on Mast Cells in Mouse Allergic Asthma. Eur J Pharmacol (2015) 754:199–210. doi: 10.1016/j.ejphar.2015.02.023 25704619

[41] Piconese S Gri G Tripodo C Musio S Gorzanelli A Frossi B. Mast Cells Counteract Regulatory T-Cell Suppression Through Interleukin-6 and OX40/OX40L Axis Toward Th17-Cell Differentiation. Blood (2009) 114(13):2639–48. doi: 10.1182/blood-2009-05-220004 19643985

[42] Molin D Edstrom A Glimelius I Glimelius B Nilsson G Sundstrom C. Mast Cell Infiltration Correlates With Poor Prognosis in Hodgkin’s Lymphoma. Br J Haematol (2002) 119(1):122–4. doi: 10.1046/j.1365-2141.2002.03768.x 12358914

[43] Fischer M Harvima IT Carvalho RF Moller C Naukkarinen A Enblad G. Mast Cell CD30 Ligand is Upregulated in Cutaneous Inflammation and Mediates Degranulation-Independent Chemokine Secretion. J Clin Invest (2006) 116(10):2748–56. doi: 10.1172/JCI24274 PMC1560346 16964309

[44] Weniger MA Tiacci E Schneider S Arnolds J Ruschenbaum S Duppach J. Human CD30+ B Cells Represent a Unique Subset Related to Hodgkin Lymphoma Cells. J Clin Invest (2018) 128(7):2996–3007. doi: 10.1172/JCI95993 29889102 PMC6025985

[45] Franco CB Chen CC Drukker M Weissman IL Galli SJ. Distinguishing Mast Cell and Granulocyte Differentiation at the Single-Cell Level. Cell Stem Cell (2010) 6(4):361–8. doi: 10.1016/j.stem.2010.02.013 PMC2852254 20362540

[46] Santos DD Hatjiharissi E Tournilhac O Chemaly MZ Leleu X Xu L. CD52 is Expressed on Human Mast Cells and is a Potential Therapeutic Target in Waldenstrom’s Macroglobulinemia and Mast Cell Disorders. Clin Lymphoma Myeloma (2006) 6(6):478–83. doi: 10.3816/CLM.2006.n.029 16796779

[47] Chen GY Tang J Zheng P Liu Y. CD24 and Siglec-10 Selectively Repress Tissue Damage-Induced Immune Responses. Science (2009) 323(5922):1722–5. doi: 10.1126/science.1168988 PMC2765686 19264983

[48] Akdis CA Blaser K. Histamine in the Immune Regulation of Allergic Inflammation. J Allergy Clin Immunol (2003) 112(1):15–22. doi: 10.1067/mai.2003.1585 12847474

[49] Jutel M Watanabe T Klunker S Akdis M Thomet OA Malolepszy J. Histamine Regulates T-Cell and Antibody Responses by Differential Expression of H1 and H2 Receptors. Nature (2001) 413(6854):420–5. doi: 10.1038/35096564 11574888

[50] Banu Y Watanabe T. Augmentation of Antigen Receptor-Mediated Responses by Histamine H1 Receptor Signaling. J Exp Med (1999) 189(4):673–82. doi: 10.1084/jem.189.4.673 PMC2192933 9989982

[51] Kimata H Fujimoto M Ishioka C Yoshida A. Histamine Selectively Enhances Human Immunoglobulin E (IgE) and IgG4 Production Induced by Anti-CD58 Monoclonal Antibody. J Exp Med (1996) 184(2):357–64. doi: 10.1084/jem.184.2.357 PMC2192716 8760789

[52] Merluzzi S Betto E Ceccaroni AA Magris R Giunta M Mion F. Mast Cells, Basophils and B Cell Connection Network. Mol Immunol (2015) 63(1):94–103. doi: 10.1016/j.molimm.2014.02.016 24671125

[53] Rogers D Vila-Leahey A Pessoa AC Oldford S Marignani PA Marshall JS. Ranitidine Inhibition of Breast Tumor Growth Is B Cell Dependent and Associated With an Enhanced Antitumor Antibody Response. Front Immunol (2018) 9:1894. doi: 10.3389/fimmu.2018.01894 30158936 PMC6104125

[54] Meghnem D Oldford SA Haidl ID Barrett L Marshall JS. Histamine Receptor 2 Blockade Selectively Impacts B and T Cells in Healthy Subjects. Sci Rep (2021) 11(1):1–10. doi: 10.1038/s41598-021-88829-w 33931709 PMC8087813

[55] Tamayo E Alvarez P Merino R. TGFbeta Superfamily Members as Regulators of B Cell Development and Function-Implications for Autoimmunity. Int J Mol Sci (2018) 19(12):3928. doi: 10.3390/ijms19123928 PMC6321615 30544541

[56] Lefrancais E Duval A Mirey E Roga S Espinosa E Cayrol C. Central Domain of IL-33 is Cleaved by Mast Cell Proteases for Potent Activation of Group-2 Innate Lymphoid Cells. Proc Natl Acad Sci USA (2014) 111(43):15502–7. doi: 10.1073/pnas.1410700111 PMC4217470 25313073

[57] Roy A Ganesh G Sippola H Bolin S Sawesi O Dagalv A. Mast Cell Chymase Degrades the Alarmins Heat Shock Protein 70, Biglycan, HMGB1, and Interleukin-33 (IL-33) and Limits Danger-Induced Inflammation. J Biol Chem (2014) 289(1):237–50. doi: 10.1074/jbc.M112.435156 PMC3879547 24257755

[58] Waern I Lundequist A Pejler G Wernersson S. Mast Cell Chymase Modulates IL-33 Levels and Controls Allergic Sensitization in Dust-Mite Induced Airway Inflammation. Mucosal Immunol (2013) 6(5):911–20. doi: 10.1038/mi.2012.129 23235745

[59] Yoshikawa T Imada T Nakakubo H Nakamura N Naito K. Rat Mast Cell Protease-I Enhances Immunoglobulin E Production by Mouse B Cells Stimulated With Interleukin-4. Immunology (2001) 104(3):333–40. doi: 10.1046/j.1365-2567.2001.01320.x PMC1783305 11722648

[60] Xue JM Yang LT Yang G Geng XR Liu ZQ Wang S. Protease-Activated Receptor-2 Suppresses Interleukin (IL)-10 Expression in B Cells *via* Upregulating Bcl2L12 in Patients With Allergic Rhinitis. Allergy (2017) 72(11):1704–12. doi: 10.1111/all.13186 28426164

[61] Marshall JS Gauldie J Nielsen L Bienenstock J. Leukemia Inhibitory Factor Production by Rat Mast Cells. Eur J Immunol (1993) 23(9):2116–20. doi: 10.1002/eji.1830230911 8370394

[62] Tumang JR Holodick NE Vizconde TC Kaku H Frances R Rothstein TL. A CD25(-) Positive Population of Activated B1 Cells Expresses LIFR and Responds to LIF. Front Immunol (2011) 2:6. doi: 10.3389/fimmu.2011.00006 22566797 PMC3342026

[63] Rosser EC Oleinika K Tonon S Doyle R Bosma A Carter NA. Regulatory B Cells are Induced by Gut Microbiota-Driven Interleukin-1beta and Interleukin-6 Production. Nat Med (2014) 20(11):1334–9. doi: 10.1038/nm.3680 25326801

[64] Breitling S Hui Z Zabini D Hu Y Hoffmann J Goldenberg NM. The Mast Cell-B Cell Axis in Lung Vascular Remodeling and Pulmonary Hypertension. Am J Physiol Lung Cell Mol Physiol (2017) 312(5):L710–21. doi: 10.1152/ajplung.00311.2016 28235950

[65] Gupta AA Leal-Berumen I Croitoru K Marshall JS. Rat Peritoneal Mast Cells Produce IFN-Gamma Following IL-12 Treatment But Not in Response to IgE-Mediated Activation. J Immunol (1996) 157(5):2123–8. 8757336

[66] Portales-Cervantes L Crump OM Dada S Liwski CR Gotovina J Haidl ID. IL-4 Enhances Interferon Production by Virus-Infected Human Mast Cells. J Allergy Clin Immunol (2020) 146(3):675–677 e5. doi: 10.1016/j.jaci.2020.02.011 32112794

[67] Domeier PP Chodisetti SB Schell SL Kawasawa YI Fasnacht MJ Soni C. B-Cell-Intrinsic Type 1 Interferon Signaling Is Crucial for Loss of Tolerance and the Development of Autoreactive B Cells. Cell Rep (2018) 24(2):406–18. doi: 10.1016/j.celrep.2018.06.046 PMC6089613 29996101

[68] Rivellese F Mauro D Nerviani A Pagani S Fossati-Jimack L Messemaker T. Mast Cells in Early Rheumatoid Arthritis Associate With Disease Severity and Support B Cell Autoantibody Production. Ann Rheum Dis (2018) 77(12):1773–81. doi: 10.1136/annrheumdis-2018-213418 30127058

[69] Syedbasha M Bonfiglio F Linnik J Stuehler C Wuthrich D Egli A. Interferon-Lambda Enhances the Differentiation of Naive B Cells Into Plasmablasts *via* the Mtorc1 Pathway. Cell Rep (2020) 33(1):108211. doi: 10.1016/j.celrep.2020.108211 33027651

[70] Oldford SA Salsman SP Portales-Cervantes L Alyazidi R Anderson R Haidl ID. Interferon Alpha2 and Interferon Gamma Induce the Degranulation Independent Production of VEGF-A and IL-1 Receptor Antagonist and Other Mediators From Human Mast Cells. Immun Inflammation Dis (2018) 6(1):176–89. doi: 10.1002/iid3.211 PMC5818443 29235261

[71] Marshall JS Portales-Cervantes L Leong E. Mast Cell Responses to Viruses and Pathogen Products. Int J Mol Sci (2019) 20(17):4241. doi: 10.3390/ijms20174241 PMC6747121 31480219

[72] Pawankar R Okuda M Yssel H Okumura K Ra C. Nasal Mast Cells in Perennial Allergic Rhinitics Exhibit Increased Expression of the Fc epsilonRI, CD40L, IL-4, and IL-13, and can Induce IgE Synthesis in B Cells. J Clin Invest (1997) 99(7):1492–9. doi: 10.1172/JCI119311 PMC507968 9119992

[73] Ahmed A Koma MK. Interleukin-33 Triggers B1 Cell Expansion and Its Release of Monocyte/Macrophage Chemoattractants and Growth Factors. Scand J Immunol (2015) 82(2):118–24. doi: 10.1111/sji.12312 25997709

[74] Wang ZZ Song J Wang H Li JX Xiao Q Yu Z. B Cell-Activating Factor Promotes B Cell Survival in Ectopic Lymphoid Tissues in Nasal Polyps. Front Immunol (2020) 11:625630. doi: 10.3389/fimmu.2020.625630 33552090 PMC7854540

[75] Ho AW Hatjiharissi E Ciccarelli BT Branagan AR Hunter ZR Leleu X. CD27-CD70 Interactions in the Pathogenesis of Waldenstrom Macroglobulinemia. Blood (2008) 112(12):4683–9. doi: 10.1182/blood-2007-04-084525 PMC2597134 18216294

[76] Skokos D Le Panse S Villa I Rousselle JC Peronet R David B. Mast Cell-Dependent B and T Lymphocyte Activation is Mediated by the Secretion of Immunologically Active Exosomes. J Immunol (2001) 166(2):868–76. doi: 10.4049/jimmunol.166.2.868 11145662

[77] Elieh Ali Komi D Grauwet K. Role of Mast Cells in Regulation of T Cell Responses in Experimental and Clinical Settings. Clin Rev Allergy Immunol (2018) 54(3):432–45. doi: 10.1007/s12016-017-8646-z 28929455

[78] Carroll-Portillo A Surviladze Z Cambi A Lidke DS Wilson BS. Mast Cell Synapses and Exosomes: Membrane Contacts for Information Exchange. Front Immunol (2012) 3:46. doi: 10.3389/fimmu.2012.00046 22566928 PMC3342342

[79] Skokos D Botros HG Demeure C Morin J Peronet R Birkenmeier G. Mast Cell-Derived Exosomes Induce Phenotypic and Functional Maturation of Dendritic Cells and Elicit Specific Immune Responses *In Vivo* . J Immunol (2003) 170(6):3037–45. doi: 10.4049/jimmunol.170.6.3037 12626558

[80] Byrne SN Limón-Flores AY Ullrich SE. Mast Cell Migration From the Skin to the Draining Lymph Nodes Upon Ultraviolet Irradiation Represents a Key Step in the Induction of Immune Suppression. J Immunol (2008) 180(7):4648–55. doi: 10.4049/jimmunol.180.7.4648 PMC2391302 18354188

[81] He S Xie H. Modulation of Tryptase Release From Human Tonsil Mast Cells by Protease Inhibitors. Pharmacol Rep (2005) 57(4):523–30. 16129920

[82] He SH Xie H Fu YL. Activation of Human Tonsil and Skin Mast Cells by Agonists of Proteinase Activated Receptor-2. Acta Pharmacol Sin (2005) 26(5):568–74. doi: 10.1111/j.1745-7254.2005.00079.x PMC7091817 15842775

[83] Kawanishi H Ihle JM. *In Vitro* Induction and Characterization of Mast Cells From Murine Peyer’s Patches. Scand J Immunol (1987) 25(2):109–20. doi: 10.1111/j.1365-3083.1987.tb01053.x 2434985

[84] Zhai GT Wang H Li JX Cao PP Jiang WX Song J. IgD-Activated Mast Cells Induce IgE Synthesis in B Cells in Nasal Polyps. J Allergy Clin Immunol (2018) 142(5):1489–1499 e23. doi: 10.1016/j.jaci.2018.07.025 30102935

[85] Rivellese F Nerviani A Rossi FW Marone G Matucci-Cerinic M de Paulis A. Mast Cells in Rheumatoid Arthritis: Friends or Foes? Autoimmun Rev (2017) 16(6):557–63. doi: 10.1016/j.autrev.2017.04.001 28411167

[86] Tkaczyk C Villa I Peronet R David B Chouaib S Mecheri S. *In Vitro* and *In Vivo* Immunostimulatory Potential of Bone Marrow-Derived Mast Cells on B- and T-Lymphocyte Activation. J Allergy Clin Immunol (2000) 105(1 Pt 1):134–42. doi: 10.1016/S0091-6749(00)90188-X 10629463

[87] Tkaczyk C Frandji P Botros HG Poncet P Lapeyre J Peronet R. Mouse Bone Marrow-Derived Mast Cells and Mast Cell Lines Constitutively Produce B Cell Growth and Differentiation Activities. J Immunol (1996) 157(4):1720–8. 8759761

[88] Palm A-KE Garcia-Faroldi G Lundberg M Pejler G Kleinau S. Activated Mast Cells Promote Differentiation of B Cells Into Effector Cells. Sci Rep (2016) 6:20531. doi: 10.1038/srep20531 26847186 PMC4742803

[89] Chacon-Salinas R Limon-Flores AY Chavez-Blanco AD Gonzalez-Estrada A Ullrich SE. Mast Cell-Derived IL-10 Suppresses Germinal Center Formation by Affecting T Follicular Helper Cell Function. J Immunol (2011) 186(1):25–31. doi: 10.4049/jimmunol.1001657 21098222 PMC3059502

[90] Rojas OL Probstel AK Porfilio EA Wang AA Charabati M Sun T. Recirculating Intestinal IgA-Producing Cells Regulate Neuroinflammation *via* IL-10. Cell (2019) 177(2):492–3. doi: 10.1016/j.cell.2019.03.037 30951673

[91] Yanaba K Yoshizaki A Asano Y Kadono T Tedder TF Sato S. IL-10-Producing Regulatory B10 Cells Inhibit Intestinal Injury in a Mouse Model. Am J Pathol (2011) 178(2):735–43. doi: 10.1016/j.ajpath.2010.10.022 PMC3069829 21281806

[92] Ahn A Park CJ Cho YU Jang S Seo EJ Lee JH. Clinical, Laboratory, and Bone Marrow Findings of 31 Patients With Waldenstrom Macroglobulinemia. Ann Lab Med (2020) 40(3):193–200. doi: 10.3343/alm.2020.40.3.193 31858758 PMC6933056

[93] Pappa CA Tsirakis G Stavroulaki E Kokonozaki M Xekalou A Konsolas I. Mast Cells Influence the Proliferation Rate of Myeloma Plasma Cells. Cancer Invest (2015) 33(4):137–41. doi: 10.3109/07357907.2015.1008639 25738408

[94] Hanes MR Giacomantonio CA Marshall JS. Mast Cells and Skin and Breast Cancers: A Complicated and Microenvironment-Dependent Role. Cells (2021) 10(5):986. doi: 10.3390/cells10050986 33922465 PMC8146516

[95] Jachetti E Cancila V Rigoni A Bongiovanni L Cappetti B Belmonte B. Cross-Talk Between Myeloid-Derived Suppressor Cells and Mast Cells Mediates Tumor-Specific Immunosuppression in Prostate Cancer. Cancer Immunol Res (2018) 6(5):552–65. doi: 10.1158/2326-6066.CIR-17-0385 29523597

[96] Oldford SA Haidl ID Howatt MA Leiva CA Johnston B Marshall JS. A Critical Role for Mast Cells and Mast Cell-Derived IL-6 in TLR2-Mediated Inhibition of Tumor Growth. J Immunol (2010) 185(11):7067–76. doi: 10.4049/jimmunol.1001137 21041732

[97] Oldford SA Marshall JS. Mast Cells as Targets for Immunotherapy of Solid Tumors. Mol Immunol (2015) 63(1):113–24. doi: 10.1016/j.molimm.2014.02.020 24698842

[98] Fischer M Juremalm M Olsson N Backlin C Sundström C Nilsson K. Expression of CCL5/RANTES by Hodgkin and Reed-Sternberg Cells and its Possible Role in the Recruitment of Mast Cells Into Lymphomatous Tissue. Int J Cancer (2003) 107(2):197–201. doi: 10.1002/ijc.11370 12949794

[99] Rabenhorst A Schlaak M Heukamp LC Forster A Theurich S von Bergwelt-Baildon M. Mast Cells Play a Protumorigenic Role in Primary Cutaneous Lymphoma. Blood (2012) 120(10):2042–54. doi: 10.1182/blood-2012-03-415638 22837530

[100] Pemberton AD Wright SH Knight PA Miller HR. Anaphylactic Release of Mucosal Mast Cell Granule Proteases: Role of Serpins in the Differential Clearance of Mouse Mast Cell Proteases-1 and -2. J Immunol (2006) 176(2):899–904. doi: 10.4049/jimmunol.176.2.899 16393974

[101] Knight PA Wright SH Lawrence CE Paterson YY Miller HR. Delayed Expulsion of the Nematode Trichinella Spiralis in Mice Lacking the Mucosal Mast Cell-Specific Granule Chymase, Mouse Mast Cell Protease-1. J Exp Med (2000) 192(12):1849–56. doi: 10.1084/jem.192.12.1849 PMC2213497 11120781

[102] Woodbury RG Miller HR Huntley JF Newlands GF Palliser AC Wakelin D. Mucosal Mast Cells are Functionally Active During Spontaneous Expulsion of Intestinal Nematode Infections in Rat. Nature (1984) 312(5993):450–2. doi: 10.1038/312450a0 6504156

[103] Vukman KV Lalor R Aldridge A O'Neill SM. Mast Cells: New Therapeutic Target in Helminth Immune Modulation. Parasite Immunol (2016) 38(1):45–52. doi: 10.1111/pim.12295 26577605

[104] Yasuda K Nakanishi K. Host Responses to Intestinal Nematodes. Int Immunol (2018) 30(3):93–102. doi: 10.1093/intimm/dxy002 29346656

[105] Hepworth MR Maurer M Hartmann S. Regulation of Type 2 Immunity to Helminths by Mast Cells. Gut Microbes (2012) 3(5):476–81. doi: 10.4161/gmic.21507 PMC3467025 22892692

[106] Nawa Y Miller HR Hall E Jarrett EE. Adoptive Transfer of Total and Parasite-Specific IgE Responses in Rats Infected With Nippostrongylus Brasiliensis. Immunology (1981) 44(1):119–23. PMC1555133 7275179

[107] Oliveira FL Bernardes ES Brand C dos Santos SN Cabanel MP Arcanjo KD. Lack of Galectin-3 Up-Regulates IgA Expression by Peritoneal B1 Lymphocytes During B Cell Differentiation. Cell Tissue Res (2016) 363(2):411–26. doi: 10.1007/s00441-015-2203-y 26003178

[108] Matsumoto M Sasaki Y Yasuda K Takai T Muramatsu M Yoshimoto T. IgG and IgE Collaboratively Accelerate Expulsion of Strongyloides Venezuelensis in a Primary Infection. Infect Immun (2013) 81(7):2518–27. doi: 10.1128/IAI.00285-13 PMC3697603 23630966

[109] Lu F Huang S. The Roles of Mast Cells in Parasitic Protozoan Infections. Front Immunol (2017) 8:363. doi: 10.3389/fimmu.2017.00363 28428784 PMC5382204

[110] Naqvi N Ahuja K Selvapandiyan A Dey R Nakhasi H Puri N. Role of Mast Cells in Clearance of Leishmania Through Extracellular Trap Formation. Sci Rep (2017) 7(1):13240. doi: 10.1038/s41598-017-12753-1 29038500 PMC5643406

[111] Kannen V Sakita JY Carneiro ZA Bader M Alenina N Teixeira RR. Mast Cells and Serotonin Synthesis Modulate Chagas Disease in the Colon: Clinical and Experimental Evidence. Dig Dis Sci (2018) 63(6):1473–84. doi: 10.1007/s10620-018-5015-6 29569002

[112] Nascimento CR Andrade D Carvalho-Pinto CE Serra RR Vellasco L Brasil G. Mast Cell Coupling to the Kallikrein-Kinin System Fuels Intracardiac Parasitism and Worsens Heart Pathology in Experimental Chagas Disease. Front Immunol (2017) 8:840. doi: 10.3389/fimmu.2017.00840 28824610 PMC5539176

[113] Pinheiro SW Rua AM Etchebehere RM Cancado CG Chica JE Lopes ER. Morphometric Study of the Fibrosis and Mast Cell Count in the Circular Colon Musculature of Chronic Chagas Patients With and Without Megacolon. Rev Soc Bras Med Trop (2003) 36(4):461–6. doi: 10.1590/S0037-86822003000400005 12937722

[114] Bannert N Farzan M Friend DS Ochi H Price KS Sodroski J. Human Mast Cell Progenitors Can be Infected by Macrophagetropic Human Immunodeficiency Virus Type 1 and Retain Virus With Maturation *In Vitro* . J Virol (2001) 75(22):10808–14. doi: 10.1128/JVI.75.22.10808-10814.2001 PMC114662 11602722

[115] Oymar K Halvorsen T Aksnes L. Mast Cell Activation and Leukotriene Secretion in Wheezing Infants. Relation to Respiratory Syncytial Virus and Outcome. Pediatr Allergy Immunol (2006) 17(1):37–42. doi: 10.1111/j.1399-3038.2005.00345.x 16426253

[116] Rabelo K Goncalves A Souza LJ Sales AP Lima SMB Trindade GF. Zika Virus Infects Human Placental Mast Cells and the HMC-1 Cell Line, and Triggers Degranulation, Cytokine Release and Ultrastructural Changes. Cells (2020) 9(4):975. doi: 10.3390/cells9040975 PMC7227014 32316163

[117] Kulka M Alexopoulou L Flavell RA Metcalfe DD. Activation of Mast Cells by Double-Stranded RNA: Evidence for Activation Through Toll-Like Receptor 3. J Allergy Clin Immunol (2004) 114(1):174–82. doi: 10.1016/j.jaci.2004.03.049 15241362

[118] Graham AC Hilmer KM Zickovich JM Obar JJ. Inflammatory Response of Mast Cells During Influenza A Virus Infection is Mediated by Active Infection and RIG-I Signaling. J Immunol (2013) 190(9):4676–84. doi: 10.4049/jimmunol.1202096 PMC3633673 23526820

[119] Brown MG King CA Sherren C Marshall JS Anderson R. A Dominant Role for FcgammaRII in Antibody-Enhanced Dengue Virus Infection of Human Mast Cells and Associated CCL5 Release. J Leukoc Biol (2006) 80(6):1242–50. doi: 10.1189/jlb.0805441 16940332

[120] Brown MG Huang YY Marshall JS King CA Hoskin DW Anderson R. Dramatic Caspase-Dependent Apoptosis in Antibody-Enhanced Dengue Virus Infection of Human Mast Cells. J Leukoc Biol (2009) 85(1):71–80. doi: 10.1189/jlb.0308167 18809735

[121] Brown MG Hermann LL Issekutz AC Marshall JS Rowter D Al-Afif A. Dengue Virus Infection of Mast Cells Triggers Endothelial Cell Activation. J Virol (2011) 85(2):1145–50. doi: 10.1128/JVI.01630-10 PMC3019992 21068256

[122] Furuta T Murao LA Lan NT Huy NT Huong VT Thuy TT. Association of Mast Cell-Derived VEGF and Proteases in Dengue Shock Syndrome. PloS Negl Trop Dis (2012) 6(2):e1505. doi: 10.1371/journal.pntd.0001505 22363824 PMC3283553

[123] Sherif NA Zayan AH Elkady AH Ghozy S Ahmed AR Omran ES. Mast Cell Mediators in Relation to Dengue Severity: A Systematic Review and Meta-Analysis. Rev Med Virol (2020) 30(1):e2084. doi: 10.1002/rmv.2084 31709696

[124] St John AL. Influence of Mast Cells on Dengue Protective Immunity and Immune Pathology. PloS Pathog (2013) 9(12):e1003783. doi: 10.1371/journal.ppat.1003783 24367254 PMC3868513

[125] Ricke DO. Two Different Antibody-Dependent Enhancement (ADE) Risks for SARS-CoV-2 Antibodies. Front Immunol (2021) 12:640093. doi: 10.3389/fimmu.2021.640093 33717193 PMC7943455

[126] Arvin AM Fink K Schmid MA Cathcart A Spreafico R Havenar-Daughton C. A Perspective on Potential Antibody-Dependent Enhancement of SARS-CoV-2. Nature (2020) 584(7821):353–63. doi: 10.1038/s41586-020-2538-8 32659783

[127] Sundstrom JB Ellis JE Hair GA Kirshenbaum AS Metcalfe DD Yi H. Human Tissue Mast Cells are an Inducible Reservoir of Persistent HIV Infection. Blood (2007) 109(12):5293–300. doi: 10.1182/blood-2006-11-058438 PMC1890823 17351109

[128] Marone G de Paulis A Florio G Petraroli A Rossi FW Triggiani M. Are Mast Cells MASTers in HIV-1 Infection? Int Arch Allergy Immunol (2001) 125(2):89–95. doi: 10.1159/000053802 11435725

[129] Nakamura Y Oscherwitz J Cease KB Chan SM Munoz-Planillo R Hasegawa M. Staphylococcus Delta-Toxin Induces Allergic Skin Disease by Activating Mast Cells. Nature (2013) 503(7476):397–401. doi: 10.1038/nature12655 24172897 PMC4090780

[130] Czarnowicki T Gonzalez J Bonifacio KM Shemer A Xiangyu P Kunjravia N. Diverse Activation and Differentiation of Multiple B-Cell Subsets in Patients With Atopic Dermatitis But Not in Patients With Psoriasis. J Allergy Clin Immunol (2016) 137(1):118–129 e5. doi: 10.1016/j.jaci.2015.08.027 26441226

[131] Schlievert PM Gourronc FA Leung DYM Klingelhutz AJ. Human Keratinocyte Response to Superantigens. mSphere (2020) 5(5). doi: 10.1128/mSphere.00803-20 PMC7568652 33028686

[132] Patel G Greenberger PA. Allergic Bronchopulmonary Aspergillosis. Allergy Asthma Proc (2019) 986:5–9. doi: 10.2500/aap.2019.40.4262. 31690385

[133] Jiao Q Luo Y Scheffel J Zhao Z Maurer M. The Complex Role of Mast Cells in Fungal Infections. Exp Dermatol (2019) 28(7):749–55. doi: 10.1111/exd.13907 30801834

[134] Urb M Pouliot P Gravelat FN Olivier M Sheppard DC. Aspergillus Fumigatus Induces Immunoglobulin E-Independent Mast Cell Degranulation. J Infect Dis (2009) 200(3):464–72. doi: 10.1086/600070 19527167

[135] Kauffman HF. Immunopathogenesis of Allergic Bronchopulmonary Aspergillosis and Airway Remodeling. Front Biosci (2003) 8(5):e190–6. doi: 10.2741/990 12456379

[136] Li J-X Fan L-C Li M-H Cao W-J Xu J-F. Beneficial Effects of Omalizumab Therapy in Allergic Bronchopulmonary Aspergillosis: A Synthesis Review of Published Literature. Respir Med (2017) 122:33–42. doi: 10.1016/j.rmed.2016.11.019 27993289

[137] Selander C Engblom C Nilsson G Scheynius A Andersson CL. TLR2/MyD88-Dependent and-Independent Activation of Mast Cell IgE Responses by the Skin Commensal Yeast Malassezia Sympodialis. J Immunol (2009) 182(7):4208–16. doi: 10.4049/jimmunol.0800885 19299719

[138] Lamoureux J Stankova J Rola-Pleszczynski M. Leukotriene D4 Enhances Immunoglobulin Production in CD40-Activated Human B Lymphocytes. J Allergy Clin Immunol (2006) 117(4):924–30. doi: 10.1016/j.jaci.2005.12.1329 16630953

[139] Khosravi AR Bandghorai AN Moazzeni M Shokri H Mansouri P Mahmoudi M. Evaluation of Candida Albicans Allergens Reactive With Specific IgE in Asthma and Atopic Eczema Patients. Mycoses (2009) 52(4):326–33. doi: 10.1111/j.1439-0507.2008.01599.x 18705661

[140] Nissen D Petersen LJ Esch R Svejgaard E Skov PS Poulsen LK. IgE-Sensitization to Cellular and Culture Filtrates of Fungal Extracts in Patients With Atopic Dermatitis. Ann Allergy Asthma Immunol (1998) 81(3):247–55. doi: 10.1016/S1081-1206(10)62821-9 9759803

